# The current research status and strategies employed to modify food-derived bioactive peptides

**DOI:** 10.3389/fnut.2022.950823

**Published:** 2022-09-02

**Authors:** Julieth Joram Majura, Wenhong Cao, Zhongqin Chen, Kyi Kyi Htwe, Wan Li, Ran Du, Pei Zhang, Huina Zheng, Jialong Gao

**Affiliations:** ^1^College of Food Science and Technology, Guangdong Ocean University, Zhanjiang, China; ^2^Guangdong Provincial Key Laboratory of Aquatic Products Processing and Safety, Guangdong Provincial Engineering Technology Research Center of Seafood, Zhanjiang, China; ^3^National Research and Development Branch Center for Shellfish Processing, Zhanjiang, China; ^4^Guangdong Province Engineering Laboratory for Marine Biological Products, Key Laboratory of Advanced Processing of Aquatic Product of Guangdong Higher Education Institution, Zhanjiang, China; ^5^Collaborative Innovation Center of Seafood Deep Processing, Dalian Polytechnic University, Dalian, China

**Keywords:** food-derived bioactive peptides, inherent drawbacks, bioactivity, modification, functional foods, therapeutic drugs

## Abstract

The ability of bioactive peptides to exert biological functions has mainly contributed to their exploitation. The exploitation and utilization of these peptides have grown tremendously over the past two decades. Food-derived peptides from sources such as plant, animal, and marine proteins and their byproducts constitute a more significant portion of the naturally-occurring peptides that have been documented. Due to their high specificity and biocompatibility, these peptides serve as a suitable alternative to pharmacological drugs for treating non-communicable diseases (such as cardiovascular diseases, obesity, and cancer). They are helpful as food preservatives, ingredients in functional foods, and dietary supplements in the food sector. Despite their unique features, the application of these peptides in the clinical and food sector is to some extent hindered by their inherent drawbacks such as toxicity, bitterness, instability, and susceptibility to enzymatic degradation in the gastrointestinal tract. Several strategies have been employed to eliminate or reduce the disadvantages of peptides, thus enhancing the peptide bioactivity and broadening the opportunities for their applications. This review article focuses on the current research status of various bioactive peptides and the strategies that have been implemented to overcome their disadvantages. It will also highlight future perspectives regarding the possible improvements to be made for the development of bioactive peptides with practical uses and their commercialization.

## Introduction

Among the bioactive substances, bioactive peptides, including those of food origin, exert the ability to influence human health (1) positively and have caught the spotlight as potential bioregulators and their utilization in food, cosmetics, and clinical field, has been massive since their discovery. Food-derived peptides are produced *in vivo* or *in vitro* from plant, insect, animal, or marine proteins. Thousands of bioactive peptides with different functionalities have been isolated from food proteins.

Examples are listed in Table 1. According to PlantPepDB, there are 3,848 plant-based peptides, among which 2,821 have been studied at the protein level (1). Plants such as oats, rice, sorghum, barley, and wheat (11, 12), legumes such as beans, peas, and lentils (13–15), mushrooms (16), nuts (17), vegetables such as broccoli (18), and fruits (19) are primary plant sources of bioactive peptides. Over 253 peptides have been identified from various marine sources (20), including fish (several species, e.g., tilapia, carp mackerel), crustaceans, and algae (21, 22). Marine peptides can also be derived from fish byproducts such as skin, viscera, scales, and heads (23, 24), usually discarded into the environment, causing pollution. The utilization of marine peptides is gaining more popularity, for instance, in the cosmetics industry, due to their anti-aging and anti-inflammatory properties (25, 26). Bioactive peptides from dairy sources have been widely exploited and isolated from milk, meat, cheese, and eggs, among other animal products (27, 28) and also from byproducts. Due to their experimentally proven health claims, functional foods and supplements containing these peptides (based on the bioactivity of interest) are commercially available. An example of such a product is a dietary supplement, “Bioactive Milk Peptides” containing casein decapeptide intended for stress relief and help with sleep.

**Table 1:** Sources of bioactive peptides from food-derived proteins.

**Source**	**Protein**	**Production process**	**Identification tool**	**Peptide/sequence**	**Potential activity**	**Reference**
Dairy	Sheep whey	Enzymatic hydrolysis: trypsin, papain, alcalase	*In silico* docking using Autodock Vina software	RLYLHENK (RL8) MQEHFTCCR (MQ9)	Dipeptidyl peptidase-IV inhibitor (DPP-IV)	(2)
	Goat whey, casein	Alcalase-assisted fermentation by Lactiplantibacillus plantarum L60 and Lacticaseibacillus rhamnosus LR22	liquid chromatography–tandem mass spectrometry (LC–MS/MS)	FFDDK, NMAHIPR, SCQDQPTTLAR	Angiotensin-1-converting enzyme inhibitior (ACE) and antioxidant	(3)
	Camel and bovine casein	Simulated gastrointestinal digestion	LCMS QTOF	FLWPEYGAL, ACGP, HLPGRG, GPAHCLL	Antidiabetic	(4)
Plant	Kiwicha	*In vitro* gastrointestinal digestion	(LC–MS/MS)	FLISCLL, SVFDEELS and DFIILE	ACE & DPP-IV inhibition, antioxidant	(5)
	Yam (*D. cayennensis*)	*In vitro* gastrointestinal digestion	nanoLC-ESI-MS/MS, MALDI-TOF-MS, *in silico* analysis done using PEAKS Studio 8.5 software, BIOPEP,	DDCAY, LLTW, LAPLPL, QLVHESQDQKR, LRPEW among others.	Antimicrobial, antioxidant effect, ACE inhibition and DNA protection.	(6)
	Adzuki Bean	Simulated digestion	Liquid chromatography-tandem mass spectrometry (UPLC-MS/MS)	KQSESHFVDAQPEQQQR	Anti-inflammatory	(7)
Marine	Rainbow trout	Alcalase-hydrolysis, simulated digestion		NI	ACE inhibitor, antioxidant	(8)
	Nile tilapia	Trypsin digestion	Molecular docking	GPEGPAGAR & GETGPAGPAGAAGPAGPR	ACE- inhibitor	(9)
	Marine snail	Enzymatic hydrolysis	nano-LC-ESI-MS/MS	YSQLENEFDR YIAEDAER	ACE- inhibitor, antioxidant, antidiabetic	(10)

Bioactive peptides are diverse in functionality, conformity, length, and size. The amino acid composition and sequence determine the peptides' physiological function. These peptides can be classified as antimicrobial (antifungal, antibacterial) (29, 30), anti-inflammatory (31, 32), anticancer (33), antihypertensive (34, 35), immunomodulatory (36), mineral-binding (37), opioids (38), and antidiabetic (39–41), based on the experimentally determined functionality. It has been reported that some peptides are multifunctional; thus, they can exhibit more than one biofunction (5, 42).

Based on the size, the majority of these bioactive peptides are of small size, typically consisting of 2–20 amino acid residues and a molecular weight of <6000Da (43). For instance, the antioxidant peptides are generally small in size (<1000Da), consisting of 5–16 amino acids per chain, with hydrophobic amino acids making a more significant proportion of their composition, contributing to higher antioxidant activity (44–46). Antihypertensive peptides vary significantly in length and are classified as tiny, small, medium, and large peptides based on the number of amino acid residues (47). However, other peptides, such as the antimicrobial peptides, may consist of up to 30- 100 amino acid residues (48, 49); an example of such is PR-39, a proline-rich antibacterial peptide derived from the pig intestine consisting of 39 amino acids (50). Several review articles on food-derived bioactive peptides are available, providing in-depth information on their production, purification, biological functions, and mechanism of action (51–55).

Peptides are inactive within their parental protein, thus requiring hydrolysis of the protein for their release. Peptide isolation from the native protein is widely conducted using conventional approaches such as enzymatic hydrolysis and microbial fermentation, with reports that they are safe (56). Several reviews have documented the production process of bioactive peptides, their purification, and analysis (51, 57). Enzymatic hydrolysis, as the name suggests this approach uses proteases (such as papain, bromelain, pepsin, trypsin, and chymotrypsin) to hydrolyze the parental protein, discharging the peptides of interest (58). Microbial fermentation involves using microorganisms capable of producing enzymes such as bacterial cultures of *Bacillus subtilis and Lactobacillus plantarum* to induce protein cleavage (59–61). Technology advancement has made the isolation of peptides from their parent protein possible through *in vitro* simulated gastrointestinal digestion, which involves using models that mimic the human digestion system to isolate peptides (62–64). Recombinant DNA technology uses microbial cells (E.coli is the most preferred since it is easy to culture and has been well characterized) for peptide yield by adding the peptide into the food matrix (65, 66).

Compared to the existing bioactive peptides that have been documented and proved to have potential benefits, it is noteworthy to mention that the translation of peptides into commercial products is still lagging. Only a few peptide-based products for human use are available on the market. The significant challenges hindering their translation are their inherent drawbacks (including toxicity, bitterness, instability, and susceptibility to enzymatic degradation in the gastrointestinal tract), regulatory obstacles, and higher production costs (67, 68). Numerous strategies have been applied to produce modified peptides, such as improved activity, reduced toxicity, and increased stability, thus subduing the drawbacks. Such techniques include modification of the peptide backbone: either by (1) the substitution of the amino acid residues, (2) insertion of new fragments, or (3) synthesis of peptidomimetics with similar bioactivity of a particular peptide of interest; microencapsulation, use of delivery systems for the release of peptides to the target site, assembly of peptides into supramolecular structures. This review highlights the research status of food-derived peptides and discusses how different techniques have been applied to overcome the drawbacks native to these peptides. The food-derived peptides reported in this review are those mainly utilized in the food and medical fields, with the ability to exert antimicrobial, antidiabetic, and antioxidant bioactivities, among others. In this article, the term modified peptides are referred to as the ones that have been altered to demonstrate improved properties: reduced toxicity, increased bioactivity, sensory quality, and stability.

## Research status of bioactive peptides

**Figure 1:**
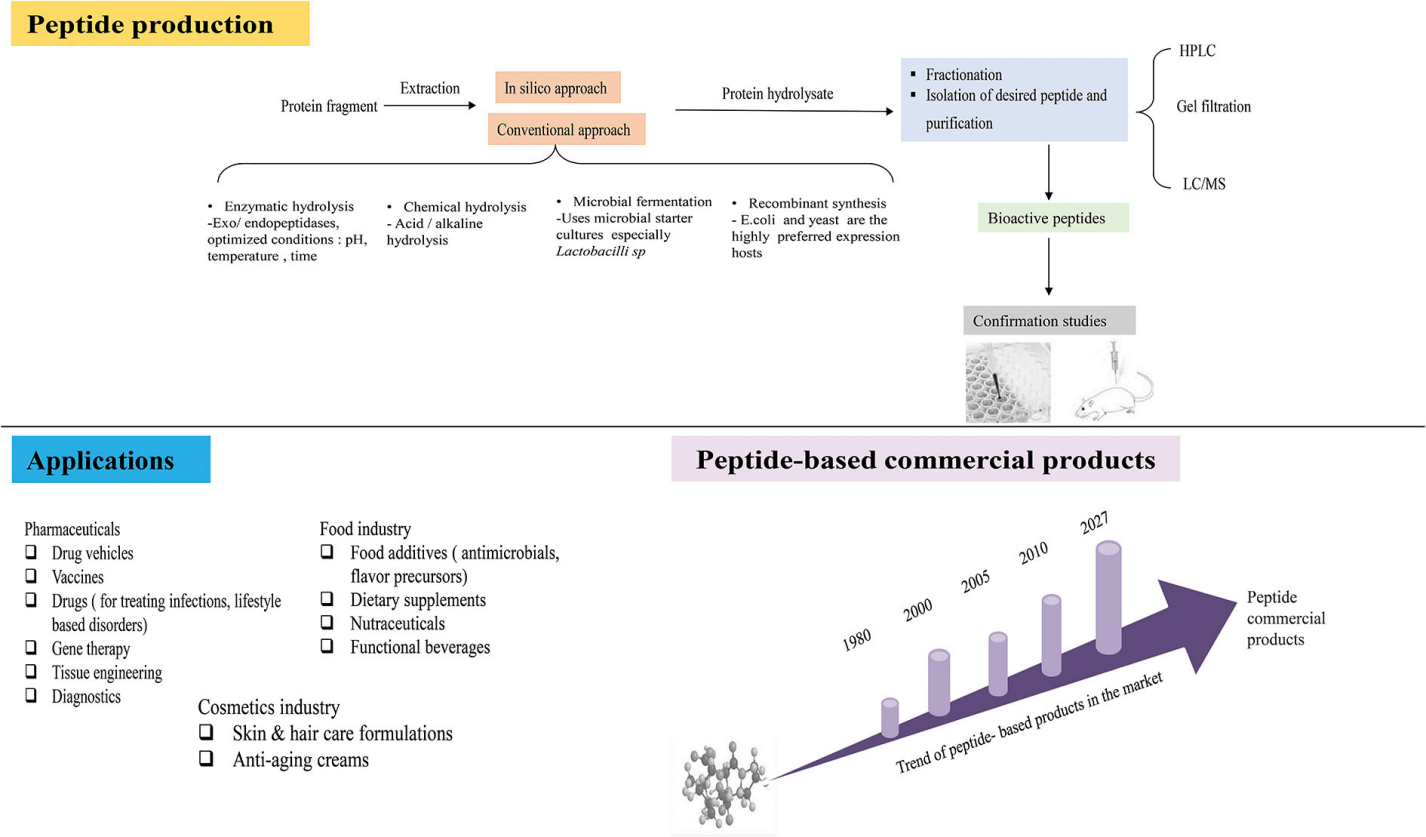
Illustration showing the experimental flow of peptide production, application of peptides and trend of commercial peptide products.

Since their discovery, bioactive peptides have attracted many researchers and have garnered widespread acceptance. They have found several applications in the food processing, cosmetics, and pharmaceutical areas, as shown in the illustration below (Figure 1), due to their exceptional ability to exert physiological function(s).

They offer several benefits, such as food preservatives, dietary supplements, and functional foods in the food sector (69, 70), and some are commercially available in the market. For instance, in 2020, BASF launched PeptAlde4.0, a rice-derived peptide anti-inflammatory product, and intends to launch two more products: PeptiStrong and PeptiYouth, derived from fava beans and peas, respectively, later this year (https://agfundernews.com/nuritas-raises-45-million-to-scale-its-plant-based-peptides-discovery-platform) [Accessed on April 20, 2022], Creatine PepForm® Peptides, a whey protein derived food supplement for enhancing muscle mass. Other studies have reported that peptides from food proteins such as soybean, milk, wheat germ, peanut, and sesame can be promising carriers for zinc supplements (71), replacing the salt inorganic derived supplements, in particular, zinc sulfate, which causes inflammation of the gastrointestinal tract (72). Nanomaterials fabricated from food-derived peptides have been applied in food emulsions (73, 74), act as building blocks for hydrogels (75), and systems for delivery and improvement of the physiochemical properties of functional compounds (76–78).

**Figure 2:**
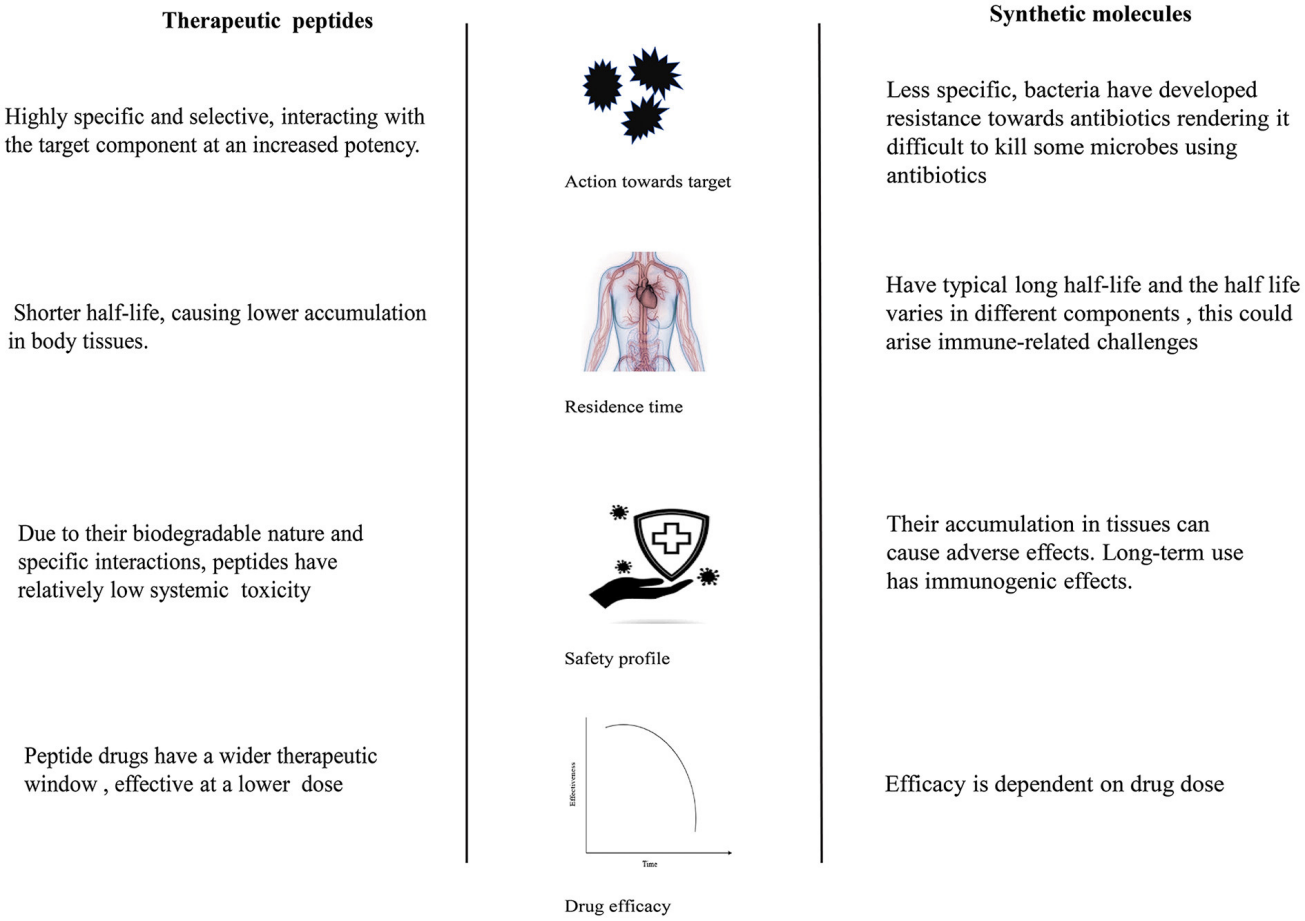
The advantages of therapeutic peptides over synthetic drugs.

In the therapeutic sector, these peptides are used as leads for drug design and alternatives to conventional drugs in treating several non-communicable/lifestyle disorders, such as obesity and diabetes, and cardiovascular and infectious diseases (79). The key characteristics that foster their translation into drugs are their high specificity, low toxicity, and ability to effectively interact with biological targets (challenging to treat with small molecules) (80) (see Figure 2). Most synthetic drugs in the market have been documented to have detrimental effects on human health. For instance, phentermine, liraglutide, bupropion/and naltrexone intended for anti-obesity have side effects; thus, their use is restricted (81). These effects leave room for the design and adaptation of peptide-based drugs because they are considered less harmful. After acting on target molecules, peptides usually disappear rapidly by proteolytic degradation, their byproducts amino acids with little toxicity (82).

The growing knowledge about the functions of peptides, increasing public awareness, and acceptance of health-promoting bioactive substances and the prevalence of diseases have boosted the global market for bioactive peptides, with North America, Europe, and the Asia Pacific being the central market (https://www.verifiedmarketresearch.com) [Accessed April 20, 2022]. Approved peptide drugs to treat several diseases are now common in the market. Peptide-based drugs account for a remarkable proportion of the pharmaceutical market income. According to the Verified Market Research (VMR) report, the revenue was valued at $48.62 billion by 2017 (https://www.verifiedmarketresearch.com) [Accessed April 20, 2022] and predicted to earn vast profits of 388 billion by 2024 (83).

## Drawbacks of food-derived bioactive peptides

Bioactive peptides as health-enhancing components are incorporated as food ingredients, dietary supplements, and nutraceuticals (84), and lead compounds for the design of therapeutic drugs intended to promote human health by treating and reducing the risk of diseases (85, 86). Unfortunately, few peptides have been successfully translated into drugs, functional foods, nutraceuticals, and food preservatives due to factors including bitter taste, high susceptibility to degradation, and poor water solubility that hinder their intensive application and commercialization (87). Table 2 contains examples of peptides with relevant disadvantages.

**Table 2:** Examples of bioactive peptides with *in vivo* drawbacks.

**Peptide**	**Drawback(s)**	**Reference**
F2,5,12W	Poor *in vivo* stability. Susceptible to enzymatic degradation and rapid clearance after being treated with serum for 5 h	(88)
F2,5,12W	Cytotoxic toward mammalian cells	(89)
BMAP-28	Cytotoxic activity against the human cells human red blood cells (hRBCs) and 3T3 cells	(90)
Piscidin-1 (fish-derived AMPs)	Extreme cytotoxic hemolysis of red blood cells	(91)

### Relatively lower activity

Generally, peptides have lower activity *in vivo*. Lower activity could be partly due to their inability to effectively penetrate the target cell and exhibit their action in the cellular environment. Cell permeability is an essential factor in designing therapeutic agents if the molecule is intended to target a component within the cell (92). Peptides are membrane-impermeable; thus, they cannot cross the cellular membrane easily. Impermeability limits their efficacy to the extracellular or transmembrane space. Their high molecular weight and polarity (due to multiple hydrogen bonding donors/acceptors in the peptide backbone) account for their impermeability (93, 94). For instance, some studies have demonstrated that a vast majority of cyclic peptides, both natural and synthetic, are cell impermeable, and their impermeability is intrinsic to the peptide structure (92).

### Poor *in vivo* stability

Peptides are naturally unstable and highly susceptible to protein degradation. Instability toward proteases in the biological systems severely hamper the translation of different bioactive peptides such as antimicrobial peptides (AMPs), antidiabetic peptides, and other therapeutic drugs. Their smaller size favors their administration *via* the oral route, the accustomed delivery route for small molecules due to patient appliance, and the lower production costs of oral drugs (95). Most peptides, especially those containing basic amino acids such as lysine and arginine, are highly prone to protease degradation in the digestive tract, tissues, and plasma (96). Upon oral administration, they can be readily hydrolyzed in acidic or enzymatic conditions causing cleavage of their amide bonds. Such cleavage causes only a tiny portion of the peptide to reach its target, resulting in low bioavailability (97–99).

Many peptides function as hormones, neurotransmitters, enzyme substrates or inhibitors, growth promoters (100), or other regulatory molecules that selectively bind to their target receptors when necessary. They can be removed rapidly when their request is expired (101), accounting for their rapid renal clearance (since the kidney usually filters out molecules below 60kDa) and a shorter half-life lasting for a few minutes, thus losing their activity (102). Also, upon administration into the human body, peptides may be susceptible to inactivation due to the concentration of salts and serum binding (103, 104), limiting their thorough clinical transformation into novel drugs.

### Safety concerns and customer preference

Since they are isolated from food proteins, most food-derived peptides are considered safe and insignificantly toxic, but this concept is still a mystery, and available evidence of their safety is scarce. Some peptides may induce toxicity and allergenicity to a certain degree (105). Bioactive peptides with toxic nature can be produced at various stages: during protein extraction, pre-treatment, hydrolysis, or upon intake (106). There are also chances for immunological uncertainties due to the complex interactions of the peptides with the host environment (107). Intestinal wall disruption, lymphocyte toxicity, production of free radicals, cytotoxicity, and immunopathic tissue damage are the major problems linked with using peptides in the biological system (108).

To meet consumers acceptance, taste is critical in developing functional foods, including peptide-based foods (68, 109). Some peptides have a bitter taste upon oral consumption (110). The bitterness property of peptides could be produced during enzymatic hydrolysis of the protein hydrolysates (111), especially those containing amino acid residues with hydrophobic side chains (109). The bitter taste reduces the peptides' sensory quality, limiting the market opportunities despite their potential health benefits.

Alternative delivery approaches such as subcutaneous and intramuscular injections are used to administer peptide-based drugs to avoid biological barriers such as proteases. This route still has limitations, including a shorter *in vivo* half-life of the drugs, thus demanding multiple injections a day, causing discomfort and poor patient adherence (112).

## Strategies employed to modify bioactive peptides

Peptide-based products legally proved and commercially available are still few despite their potential benefits. As discussed in the previous section, several challenges limit their thorough commercialization. Several strategies have been employed to overcome the obstacles. Some techniques have focused on modifying the peptide's amino acid composition (such as substituting L- with D- amino acids), terminal regions, or entrapping peptides into delivery systems resulting in novel peptide analogs with higher activity, stability, bioavailability, and reduced toxicity. Numerous bioactive peptides of different natural protein sources, including food, have been modified in this manner (113).

### Peptide backbone modification

Several bioactive peptides have been modified *via* selectively adjusting the peptide backbone. Some synthetic analogs of natural peptides have also been designed and produced in this manner. For example, the novel antimicrobial peptide L10 (WFRKQLKW) developed from the amino acid substitution of the N-terminal domain of bovine lactoferrin (114). Modifying the primary amino acid sequence can improve the overall activity, stability, and selectivity and minimize the toxicity of bioactive peptides. Since only a tiny portion of crucial amino acids are responsible for its function, some modification strategies such as the substitution of the other residues with potential amino acids (for example, D- and unnatural amino acids), the extension of the peptide chain, and the introduction of essential fragments can improve the activity without hindering its primary function (115–117).

#### Amino acid substitution

Amino acid substitution is one of the common strategies employed to improve the activity of bioactive peptides. One of the significant advantages of this strategy is that it does not cause any remarkable change in the peptide's secondary structure. Thus, the peptide's functionality is intact. Substitution of amino acids at specific positions within the peptide sequence may increase the resistance of the peptide toward proteolytic degradation (118).

For instance, numerous antimicrobial peptides have been modified and designed *via* this strategy, as depicted in Table 3. The functionality of antimicrobial peptides is highly dependent on their cationic nature, which allows electrostatic binding to the anionic components of the target cell membrane, hydrophobicity which prolongs the association of the peptide with the membrane, and amphipathicity, which enables bilayer penetration and disruption causing cell death (133, 134). The substitution with amino acid residues such as arginine (Arg), lysine (Lys), and tryptophan has been shown to positively influence the factors mentioned above, thus improving the activity of AMPs (134–138). In contrast, the replacement of arginine and tryptophan in the peptide chain has enhanced peptides' antimicrobial activity (see Table 3). L-amino acids, unlike D-amino acids, are easily susceptible to enzymatic degradation by proteases. Due to its spatial configuration, which is not recognized by proteases or immune receptors, Peptides with D- amino acids are characterized by increased resistance toward proteases degradation (139, 140). Previous studies demonstrated that D-substitution was a convenient technique to heighten the *in vivo* activity of antimicrobial peptides (141, 142). Substitution with bulky aromatic amino acids produces modified peptides with higher functionality and increased stability in physiological conditions, for example, salt (143). The replacement of tryptophan residues with bulky aromatic amino acids in the antimicrobial peptide FKCRRWQWRMKKLGA derived from Lactoferricin bovine (LFB) enhanced the peptide's antibacterial activity (144).

**Table 3:** Peptide modification *via* amino acid substitution.

**Target peptide**	**Substitution/Insertion**	**Advantage(s)**	**Reference**
Sushi 1 peptide from horseshoe crab hemocyte	Arginine	Broader spectrum of antibacterial activity against both and gram-negative bacteria, including the methicillin-resistant *Staphylococcus aureus*,	(119)
Amyl-1-18,	Aspartic acid with arginine	Enhanced antifungal activity against *Candida albicans*	(120)
buCATHL4B	Tryptophan with non-natural amino acid Azulenyl-Alanine	Enhanced proteolytic stability and cytocompatibility with human cells.	(121)
HPA3N-T3	Arganine and Tryptophan with lysine and leucine, respectively	Significant decrease in hemolytic activity than the native peptide	(122)
HPA3NT3-A2	l-Lysine residues with d-Lysine residues	Enhanced stability and antimicrobial activity against E. coli, S. aureus in serum,	(123)
RRWWRWWRR	Tryptophan with histidine	Increased antimicrobial activity, lower cytotoxic and hemolytic activity	(124)
WRWRW	N-terminal arginine residue with a metallocene moiety	Improved antibacterial activity	(125)
F2,5,12W	Phenylalanine → Tryptophan	Enhanced antimicrobial activity against bacteria *Bacillus anthracis* and *Yersinia pestis*, increased LPS neutralizing activity, and decreased salt sensitivity.	(88)
F2,5,12W	Insertion of cysteine	Increased plasma stability	(126)
AMP Jelleine-1	Arginine and tryptophan	Higher antimicrobial activity toward the multidrug-resistant *P. aureginosa*	(127)
CAMP	Incorporation of non-natural amino acid residues	Increased hydrophobicity and enzymatic stability	(128)
Pep05.	Substitution of L-Arg & L-Lys residues with D- and unnatural amino acids (D-Lys, D- Arg)	Significant protease resistance and acute toxicity *in vitro*	(129)
Piscidin-1	Threonine residues with lysine	Reduced cytotoxicity, higher antibacterial activity than native peptide	(130)
CPF-C1	Introduction of Lys, tryptophan, and D-amino acids	Enhanced antimicrobial activity against multidrug-resistant strains	(131)
Chicken cathelicidin-2	D-amino acid substitution	Improved serum stability	(132)

#### Chemical modification

Chemical modification of peptides has significantly improved their enzymatic stability and intestinal permeability. Conjugation of therapeutic peptides with potent macromolecules and metals seems ideal for delivering peptides and protein-based peptides and improving the pharmacokinetic properties of peptides, including their *in vivo* stability and overall activity (145). Conjugation with macromolecules may bestow peptides with increased resistance to protease hydrolysis since macromolecular chains can shield the enzymatic sites on peptides (126). Peptide-conjugates can be formed *via* attachment of the macromolecules to peptides either through cleavable or non-cleavable linkers, resulting in releasable and stable conjugates. Releasable conjugates as the term suggests, the drug is separated and released from the carrier in its native form. In comparison to stable conjugates, releasable conjugates are more effective. Loss of drug potency is a major drawbacks associated with the use of stable conjugates. The bulky PEG moiety tends to reduce drug activity, and higher concentration of the conjugate is needed so as to maintain the drug activity (146). Conjugation with albumin-binding molecules is commonly used to increase peptides' half-life and *in vivo* stability (147). Examples of such molecules include polyethylene glycol polymers (PEG), [COSAN]- and XTEN. Table 4 shows examples of chemically modified peptides.

**Table 4:** Chemical modification of food-derived bioactive peptide (s).

**Peptide**	**Chemical modifier**	**Outcome**	**Reference**
PMAP-37 (F34-R)	Cholesterol fragments binded to the N-terminal	Enhanced antibacterial and anti-biofilm activities, improved stability, wound healing activity *in vivo*	(148)
Clavanin A VFQFLGKIIHHVGNFVHGFSHVF-NH2),	Zn ^2+^	A remarkable increase in antimicrobial activity,	(149)
AWKR6	XTEN generating a potent XTENylated -AWKR6 conjugate	Prolonged plasma half-life by nearly 5-fold, higher GLP-1R-binding	(150)
Exenatide	mPEG	Improved hypoglycemic activity,	(151)
Bac7 (1–35)	PEG through a cleavable ester bond or through a non-hydrolyzable amide bond	Reduced renal clearance	(152)

#### PEGylation

PEGylation, a process that involves the addition of a polyethylene glycol chain to a biomolecule, has been the first and most frequently applied approach to enhance peptide and proteins' pharmacokinetic (PK) properties drugs for over 25 years (153). PEG polymer is FDA-approved, non-toxic, non-immunogenic, and highly water-soluble. The polyethylene glycol (PEG) moiety is typically attached to peptide and protein drugs shielding the surface and increasing its molecular size, lowering its susceptibility toward proteolytic degradation and the clearance rate *via* renal ultrafiltration. Generally, PEGylation improves the *in vivo* efficacies of these peptides and peptide-based drugs by conveying its physio-chemical characteristics to the peptides without interfering with their biological function(s) (154).

A massive trend in the development of PEGylated drugs has been observed since the launch of ADAGEN (pegademase bovine), the first approved PEGylated protein manufactured by Enzon Pharmaceuticals in 1990 (155). PEGylated protein and peptide therapeutics are available on the market, and many more are still under development and clinical trials. Over the predicted period of 2021-2026, the market for PEG protein therapeutics is expected to rise at a compound annual growth rate (CAGR) of 9.3%. The rise in the number of chronic diseases (such as kidney diseases, cancer, and rheumatoid arthritis), awareness of detrimental effects of chemo and radiotherapy treatments (for instance, in cancer treatment), and the demand for drugs with suitable pharmacological activity attribute to the rapid increase of PEG protein therapeutics (https://www.mordorintelligence.com/industry-reports/pegylated-proteins-market) [Accessed April 22, 2022].

Several reviews have discussed PEG-peptide conjugates, their advantages over native peptides, and their application in drug delivery (99, 156, 157). The majority of the PEGylated drugs on the market have shown the benefits of improved pharmacological activity such as *in vivo* stability (e.g., Cimzia, Neulasta, PegIntron, Adynovate, Oncaspar), circulation time (e.g., Eligard, Renagel), delivery to target (e.g., Eligard), extended-release and reduced toxicity (e.g., AmBisome, Albecet). However, despite the successful application of PEG in the pharmaceutical field, PEG has been known to raise some safety concerns. Zhang and colleagues have discussed the drawbacks of PEG in-depth, highlighting that it is both immunogenic and antigenic, non-biodegradable, and growing concerns about the effects of its remains (158).

#### XTENylation

The conjugation of therapeutic peptides with XTEN has been reported to exhibit an extended half-life and reduced clearance from circulation compared to the native non-conjugated peptides. XTEN is a hydrophilic and biodegradable non-structural protein-polymer designed to mimic the biophysical properties of PEG (159). XTEN offers a biodegradable alternative to PEG. It is stable in serum conditions, but unlike PEG, it can be easily degraded by proteolytic enzymes after subsequent internalization into cells, reducing the risk of kidney vacuolation resulting from continuous treatments. Also, its biodegradable nature reduces its accumulation in tissues, preventing toxicity under normal circumstances (160). So far, no evidence has been documented on the adverse effects of XTEN.

#### Modification of the terminal regions

Functionalization of the terminal regions of the peptide chain may help shield the peptidefrom proteolysis degradation, thus increasing its stability *in vivo* (161). For instance, the modifications of the extremes through acetylation of the N-terminus and transformation of the C-terminus into a primary amide can increase peptides' overall stability and activity, given that these two regions are not involved in binding interactions (94).

#### Synthetic peptidomimetics

Peptidomimetics are tiny, protein-like chains that mimic traditional peptides and retain the ability to interact with biological targets and produce a similar natural effect. They offer advantages that make them excellent candidates over the physiologically active peptides in the pharmacology field. These functional mimics circumvent the pharmacokinetic hurdles of native peptides (162, 163), such as their prolonged stability in biological matrices (164). Peptidomimetics have gained much attention, and to date, numerous mimics in the market developed, some being analogs of food-derived bioactive peptides that function as mediators and are helpful in therapy. Cyclizing the linear peptide and coupling unnatural amino acids are common preparations for peptidomimetics. Significant advancement has been attained in the past concerning the development of peptidomimetics, for instance, those that mimic the bactericidal activity and mode of action of AMPs (165).

#### Cyclization of peptides

Studies have confirmed that cyclization increases the resistance of peptides toward enzymatic degradation since the amide bond of the peptide is hidden inside the helix, thus increasing resistance toward proteases (94, 166). Cyclic peptides, as the name suggests, these peptides take up a cyclic ring structure and are formed by linking together the two side chains of the same peptide *via* a stable bond (such as amide, disulfide, ether) to maintain and stabilize the helical structure of the chain (167). For instance, stapling is a cyclization technique that provides the peptide with an external brace that limits its flexibility and improves its affinity and selectivity to the target. This closed conformation hides the amide bond inside the helix, increasing resistance toward proteases and allowing an easier permeation into the target cellular membrane (94, 166). Among the nine overall methods for synthesizing cyclic peptides, head-to-tail fashion is the most straightforward and frequently used cyclization technique in which the linear peptides are stapled *via* the C- and N- terminals (168). The application of cyclic peptides (both naturally occurring and synthetic) as novel therapeutic paradigms in the modern pharmaceutical field has proliferated. For instance, from 2006 to 2015, nine cyclic peptide drugs have been approved and are currently available in the market (https://www.biochempeg.com/article/121.html) [Accessed April 22, 2022] with a wide range of biological functions such as enzyme inhibition, antimicrobial, anticancer, and antidiabetic. Cyclic peptides are characterized by increased cell permeability, protease stability, and pharmacological activity (higher retention time in blood, oral absorption) compared to their linear counterparts (169). The absence of amino and carboxyl ends accounts for the resistance of the cyclic peptides toward exogenous proteases. The increased cell permeability of these cyclic peptides results from exposure of the hydrophobic region to the surface while the hydrophilic regions are concealed inside the structure. Despite being suitable drug-like substances, it has been reported that cyclic peptides are not generally cell-permeable compared to their liner equals and have poor oral bioavailability (170, 171).

### Extended-release technology

Excellent drug delivery systems shield the therapeutic molecule from premature degradation upon administration, enhance drug activity and reduce the occurrence of toxicity (172). Such delivery systems are the controlled release systems, which have been studied as an effective means for drug delivery compared to conventional delivery systems (capsules, tablets, ointments, granules, syrup, medicated gums). In traditional delivery, drugs are rapidly eliminated from the body; thus, a frequent dosage is required to maintain its therapeutic index, unlike in a controlled release system, whereby drugs are delivered at a specific target site and at a controlled rate while offering the intended therapeutic effect. The decrease in total dosage, such that a drug is administered weekly, monthly, or quarterly, is a significant advantage associated with the controlled release of drugs (173). Examples of delivery systems such as microcarriers, hydrogels, liposomes, and nanoparticles have been used for the controlled release of peptides into the human body, as shown in Table 5.

**Table 5:** Examples of controlled delivery systems for bioactive peptides.

**Delivery system**	**Example material**	**General features**	**Clinical application(s)**	**Drawback(s)**	**Reference**
Hydrogels	Chitosan-based, alginate-based, hyaluronic based	Three-dimensional polymer, its high affinity for water absorption gives it a resemblance to living tissues, higher compatibility to biological systems than other synthetic polymers, biodegradability	Localized drug delivery: can deliver the drug through the hostile environment of the stomach and at specific sites within the gastrointestinal tract (GI) such as the colon. Also valuable for diagnostics and tissue engineering as scaffolds.	Conventional hydrogels are associated with toxicity, challenging to sterilize, limited curative use	(174, 175)
Liposomes	siRNA	Possess the ability to capture both hydrophilic & lipophilic molecules,	Reduces systemic toxicity of peptides, efficient delivery of the peptide to its target site.	Poor stability and circulation time in the blood, rapid clearance	(176)
Nanoparticles	Acrylic-based polymers, polyanions (e.g., Eudragits), polycations (e.g., chitosan)	Stable in the GI tract	Encapsulate drugs hence protecting them from low pH conditions and enzymatic degradation		(177)
Microencapsulation (Microsphere, microcapsules, microparticles)	Hyaluronate, calcium alginate (CA)-carboxymethyl cellulose, PGLA	Good compatibility	Improves stability, target delivery of drugs	Water insolubility, anaphylactic reactions, poor mechanical strength	(178)

### Peptide self-assembly

Self-assembled peptides, both natural and synthetic, have become a popular hotspot in pharmaceutical and food processing, among other industries, due to their advantageous features such as resistance to proteolytic degradation, biocompatibility, and biodegradability (179–181). Apart from the self-assembled peptides which occur naturally in a food protein matrix (180), single peptides can be modified depending on the amino acid sequence *via* non-covalent interactions such as hydrogen bonding, aromatic stacking, and Van der Waals forces to form supramolecular nanostructures such as nanofibers, nanoparticles, hydrogels, and nanovesicles with specific properties (182, 183). This can be initiated through the chemical addition of a moiety, for instance, using protected amino groups or lipids to provide the driving force necessary to foster self-assembly (184). The activity (185), cell selectivity, and stability (186) of several antimicrobial peptides have been enhanced through self-assembly. An in-depth review of the self-assembled peptides, their types, their characteristics, and their applications has been made by several scholars (181, 187–189). Despite its remarkable features, the self-assembly of peptides is associated with some limitations, for instance, difficulty in the purification of the nanostructures, their lower stability under physiological conditions, and safety-related concerns (75). The peptide hydrogels can instigate biofilm formation, thus causing an imbalance of microorganisms in humans (190).

#### Other techniques

Among other factors, instability, bitter taste, and hygroscopicity limit the direct application of peptides in the development of functional foods (191). Studies have reported that encapsulation of peptides can overcome these challenges, thus improving peptides' sensory property, bioactivity, and stability (87). The stability of antioxidant peptides from flaxseed protein was improved *via* spray-drying encapsulation of the peptide along with the use of surfactants (192). Bitter peptides were less bitter and had improved gastrointestinal stability following their encapsulation in water-in-oil high internal phase emulsions (193). The entrapment of egg white-derived peptides into chitosan- tripolyphosphate nanoparticles improved its bioavailability (194).

The chelation of bioactive peptides with metals also enhances the activity affecting the peptide's structure, charge, and mode of action. Binding divalent metals such as copper and zinc have regulated the antimicrobial activity of the peptide. Piscidin 1& 3 peptides from fish mast cells had improved antibacterial activity, membrane insertion, and increased cytotoxicity against cancer cells when chelated with copper Cu^2+^ (195, 196). The antibacterial activity was improved upon the zinc Zn2+ to ClavaninA, demonstrating the ability to cleave the bacterial chromosomal DNA (197).

## Future perspective and conclusion

Generally, the advancement and development of technology have facilitated the research and utilization of bioactive peptides. The availability of peptide-based products: therapeutic drugs, functional foods, and additives in the modern market is a vivid outcome of the tremendous progress made. Despite their limitations, bioactive peptides have great potential. As discussed in the previous section, peptides have been modified using several methods to eliminate their disadvantages. The modification of bioactive peptides has been successful to a satisfactory level, in enhancing the bioactivity and physiochemical properties of peptides. However, more studies need to be conducted since the outlined strategies have limitations also.

Addressing the peptides' intrinsic drawbacks alone does not foster their translation into commercial products. Other hindrances, including manufacturing costs and regulatory challenges, are yet to be overcome since they impede the commercialization of peptides. Establishing universal criteria for approval of peptide-based products will expand the international market for these products. Time is yet another significant obstacle facing manufacturers since it takes a relatively long time to approve a peptide drug legally. The 10–12-year lag between a peptide drug candidate passing into clinical trials and potential approval (https://www.polypeptide.com/wp-content/uploads/2019/10/1401702726538c49464a6f5.pdf) [Accessed April 22, 2022] is somewhat challenging. Even after peptide drugs are approved for treatment, they can still be withdrawn/recalled from the market due to drug failure. Such circumstances become a burden on the manufacturers due to the costs incurred in producing the drugs. An example of this was the withdrawal from the market of Omontys (Peginasatide), a peptide drug intended to treat symptomatic anemia and related chronic kidney disease, due to reports of hypersensitive reaction associated with the drug (https://www.takeda.com/en-us/newsroom/news-releases/2014/affymax-and-takeda-announce-termination-of-omontys-peginesatide–product-collaboration-and-license-agreement/) [Accessed April 22, 2022].

Also, the information on the peptides' mode of action *in vivo*, their specific safety level, and their interaction(s) with the human systemis scarce; this calls for more investigation to be carried out. Addressing these challenges and discovering new bioactive peptides brightens the future for bioactive peptides, which could result in extensive utilization and huge market demand.

## Author contributions

JM: investigated, conceptualized, and wrote the original manuscript. WC: conceptualized, reviewed, edited, and responsible for the supervision of the entire manuscript. ZC, KH, WL, RD, and PZ: proofread and edited. HZ and JG: reviewed the final draft. All authors contributed to the article and approved the submitted version.

## Funding

This work was supported by the National Natural Science Foundation of China (32172163), the First Class Provincial Financial Special Fund Project of Guangdong Ocean University (231419014), China Agriculture Research System of MOF and MARA, and the Innovative Team Program of High Education of Guangdong Province (2021KCXTD021).

## Conflict of interest

The authors declare that the research was conducted in the absence of any commercial or financial relationships that could be construed as a potential conflict of interest.

## Publisher's note

All claims expressed in this article are solely those of the authors and do not necessarily represent those of their affiliated organizations, or those of the publisher, the editors and the reviewers. Any product that may be evaluated in this article, or claim that may be made by its manufacturer, is not guaranteed or endorsed by the publisher.
